# Development and Implementation of a Community Paramedicine Program in Rural United States

**DOI:** 10.5811/westjem.2020.7.44571

**Published:** 2020-08-24

**Authors:** Lucas A. Myers, Peter N. Carlson, Paul W. Krantz, Hannah L. Johnson, Matthew D. Will, Tasha M. Bjork, Marlene Dirkes, Justin E. Bowe, Kirk A. Gunderson, Christopher S. Russi

**Affiliations:** * Mayo Clinic, Mayo Clinic Ambulance Service, Rochester, Minnesota; † Mayo Clinic Health System, Department of Emergency Medicine, Eau Claire, Wisconsin; ‡ Mayo Clinic Health System, Department of Administration, Eau Claire, Wisconsin; § Mayo Clinic, Department of Emergency Medicine, Rochester, Minnesota

## Abstract

**Introduction:**

Community paramedicine (CP) is an innovative care model focused on medical management for patients suffering from chronic diseases or other conditions that result in over-utilization of healthcare services. Despite their value, CP care models are not widely used in United States healthcare settings. More research is needed to understand the feasibility and effectiveness of implementing CP programs. Our objective was to develop a CP program to better meet the needs of complex, high-utilizer patients in a rural setting.

**Methods:**

We conducted an observational descriptive case series in a community, 25-bed, critical access hospital and primary care clinic in a rural Wisconsin county. Multiple stakeholders from the local health system and associated ambulance service were active participants in program development and implementation. Eligible patients receiving the intervention were identified as complex or high need by a referring physician. Primary outcomes included measures of emergency department, hospital, and clinic utilization. Secondary measures included provider and patient satisfaction.

**Results:**

We characterized 32 unique patients as high utilizers requiring assistance in medical management. These patients were enrolled into the program and categorized as high utilizers requiring assistance in medical management. The median age was 76 years, and 68.8% were female. After six months, we found a statistically significant decline in patient utilization for primary care (53.3%, p = .006) and ED visits (59.3%, p = .007), but not for hospitalizations (60%, p = .13, non-significant (NS), compared to the six months preceding enrollment. Overall, the total number of healthcare contacts was increased after implementation (623 before vs 790 after, + 167, +26.8%). Implementation of the CP program resulted in increased overall use of local healthcare resources in patients referred by physicians as high utilizers.

**Conclusion:**

The implementation of an in-home CP program targeting high users of healthcare resources resulted in a decrease in utilization in the hospital, ED, and primary care settings; however, it was balanced and exceeded by the number of CP visits. CP programs align well with population health strategies and could be better leveraged to fill gaps in care and promote appropriate access to healthcare services. Further study is required to determine whether the shift in type of healthcare access reduces or increases cost.

## INTRODUCTION

Community paramedic (CP) programs are an evolving practice of non-emergency, community-based care. The traditional model of emergency medical services (EMS) in the United States focuses on the response to acute injury and illness. Conversely, the primary foci of CP programs are preventive, with an emphasis on primary care delivery, prevention, screening, and wellness. CP programs target several areas of emphasis, including patients who have difficulty managing single or multiple chronic diseases, who have high risk for readmission after discharge, and in general overuse healthcare services.^1^

In 2015 more than 100 agencies established CP programs in 33 states^2^ with variable state regulatory environments. Some states, such as Minnesota, have established a level of care and Medicaid reimbursement through legislation. However, at the time of this study, the State of Wisconsin had not passed legislation allowing for reimbursement. Therefore, the value of a CP program may best be demonstrated through reduced use of services. The aim of this study was to describe a CP program developed by a health system medical provider and an associated ambulance service and to analyze its impact on healthcare use and the subsequent financial ramifications.

## METHODS

### Setting

The primary service area for this study was a rural area in northwestern Wisconsin. The county has a population of 45,563^3^ and covers approximately 890 square miles. The area ambulance service employs nine full-time, ground-ambulance emergency medical technicians and paramedics and receives approximately 1500 combined emergent and non-emergent requests for service annually. The regional health system hospital is a 25-bed, critical access hospital and primary care clinic with 445 employees. This project was approved by the Mayo Clinic Institutional Review Board.

### Multidisciplinary Program Creation

A multidisciplinary team from the health system site and the associated ambulance service was formed in July 2015 to develop the CP program, which included the following features: emergency physician leadership; health system administration; EMS leadership and research coordination; nursing, home health and hospice care, the Office of Population Health, palliative care; quality resources (management engineering and internal consulting); information technology; compliance; and local paramedics.

The multidisciplinary team identified gaps of care within the community through quantitative data analysis and stakeholder interviews and then identified opportunities in the emergency department (ED) and the primary care department, with a focus on high-use patients. Through development of the program infrastructure, process, and procedures, the team consulted with home health and hospice services staff. Over 12 months, the team focused on the development of care processes and procedures, paramedic communication with physicians for patient care plans, an appropriate referral and scheduling process, and assessment of quality outcomes. Routine reports were presented to ambulance service and health system leaders. The group met weekly with CP personnel to discuss patient volume and issues with the program.

**Population Health Research Capsule**

What do we already know about this issue?

Community paramedicine (CP) is a flexible, relatively low cost way to extend primary care outside of the clinic and hospital setting and into the community.

What was the research question?

Does implementation of a CP program decrease utilization of traditional healthcare resources?

What was the major finding of the study?

CP decreased utilization, but was balanced and exceeded by the number of visits.

How does this improve population health?

CP is an important tool in decreasing high-cost healthcare utilization such as emergency department visits and hospitalizations.

### Medical Director

The physician who was the medical director for the CP program also served as the medical director for two local ambulance services and a local paramedic training institution in addition to working as an emergency physician within the same health system as the one in this project. The CP **program medical director** (PMO) assisted in all aspects of development and implementation and focused specifically on educating and interacting with referring physicians, developing medical guidelines, and reviewing medical records for quality purposes.

### Community Paramedicine Education

Two local CPs, chosen by the site manager, attended a CP training program at Hennepin Technical College in Eden Prairie, Minnesota.^4^ The one-semester, distance education course consisted of 72 hours of classroom time, 72 hours of online content, and 196 hours of clinical time conducted locally. The standardized curriculum was created by the North Central EMS Institute.^5^ Both CPs attended all clinical hours at the regional hospital that participated in this program, and spent considerable time with primary care physicians enrolling patients into the program. Beyond the CPs’ clinical education, the secondary intention was to create a working relationship and rapport between the CPs and the referring physicians. Additionally, clinical time was spent with staff involved with home health and hospice, wound care, the ED, respiratory therapy, and mental health. Both CPs received CP certification through the Minnesota Emergency Medical Services Regulatory Board before the start of the program. During this project, the state of Wisconsin did not offer CP certification, but it did allow CP projects to occur within the state with prior approval.

### Education for Referring Physicians

The CP PMO provided formal presentations to primary care physicians and hospitalists and informal presentations to emergency physicians. The broad inclusion criteria were intended to recruit patients who were high users of the ED and the clinic, who had a high risk for readmission or falls, who had chronic illness or needed postsurgical wound care, or who required frequent international normalized ratio (INR) monitoring or other blood tests. The only exclusion criteria were patients younger than 21 years and patients in skilled nursing facilities. An online survey to evaluate physicians’ perceptions of the program was distributed to all physicians who had referred at least one patient.

### Staffing Model

The two CPs were each allocated 20 hours weekly for a total of 40 hours per week: 10 hours for patient scheduling, administrative duties, and visit planning, and 30 hours for in-home visits. The model allotted one hour per in-home visit and 30 minutes of travel time per visit. With visits scheduled Monday through Friday, the maximum number of patient visits per week was 20 (four per day).

### Medical Guidelines Development

Medical guidelines were developed from existing CP program guidelines, with permission, from Eagle County Paramedic Services in Edwards, Colorado.^6^ The medical guidelines were adapted and used to address the specifics of a home visitation, including the history and examination, medication reconciliation, home assessment, and specific procedures such as drawing blood, point-of-care testing, and wound care. The state of Wisconsin required receipt of these protocols before the program began to ensure that all medical care was within the current, state-defined paramedic scope of care.

### Integrated Health Record

The regional health system hospital used an electronic health record (EHR), while the ambulance service used an EMS-specific product. The hospital EHR allowed for scheduling of patient visits and direct messaging between the ordering physician and the CP. Additionally, the CP required access to the hospital EHR to review the medical order and pertinent clinical history. The CPs received training to perform all documentation in the hospital EHR. Information technology specialists developed the new documents and templates within the EHR. No documentation was done within the EMS patient-record system. Each CP received a company-issued smartphone for business relating to the CP program and to support EHR documentation while in the patient’s home.

### Scheduling

Physicians identified potential candidates for the CP program during clinic appointments, ED visits, or hospital stays. If the patient agreed to enroll, the physician completed an order for a CP visit and documented objectives and a care plan in the EHR for the CP to review. The order was automatically printed in the paramedic office, and one of the CPs would schedule the visit. The physician determined the frequency of visits (eg, once weekly or twice weekly). Patient scheduling was shared with the ambulance dispatch center to allow for safety checks every 30 minutes during on-scene time. Visits occurred Monday through Friday from 8am to 5pm, with each visit lasting approximately one hour.

### Vehicle and Equipment

The CPs used a clearly marked passenger car that required no additional modification. The vehicle was equipped with a response bag containing equipment for assessment. Additional equipment that was not already carried by the ambulance service included the following: a scale and measuring tape (to measure the patient’s weight and height); an INR testing machine; an otoscope; laboratory blood vials from the hospital; and a cooler for transporting specimens. Each CP was given a laptop computer (for in-home documentation into the EHR) and a cell phone.

### Patient Visit

1. Before the patient visit, the CP accessed the patient’s EHR to review and confirm the physician’s order, care plan, history, visit notes, laboratory test results, and current medications and doses. CPs arrived at the patient’s home at the scheduled time and called the dispatch center to confirm their arrival. While meeting with the patient, the CPs focused on six key areas:
2. Present health status: evaluation of activity level; patient perception of health; and current medications.
3. Past health history: review of allergies; illnesses, surgical procedures; hospitalizations, immunizations, most recent evaluation by a physician, and family medical history.
4. Physical examination: review of general health status and specific systems.
5. Medication reconciliation: review of current medications, including dosages, daily schedule, and adherence to therapy; identification of medications that might have been prescribed by another physician or another medical provider; and assisting with sorting of medications if a sorting system was used.
6. Environmental assessment: use of the Physical Environment Assessment Tool (PEAT) scale at the first visit and at subsequent visits when necessary.^7^
7. Specific physician orders: review of specific orders, such as providing wound care; monitoring INR; testing blood glucose; or drawing blood.

The CP documented information from the assessment, including the PEAT scale, into the EHR through mobile remote access while still at the patient’s home or after the visit. If the CP had any concerns, the CP called the ordering physician or the on-call physician for direction. If any assessment finding indicated the need for urgent assessment by a physician (eg, chest pain or stroke symptoms), the CP was instructed to call 911.

### Physician Review of the Visit

After the CP completed documentation in the EHR, an automated alert was sent to the ordering physician and the CP program medical director. If a single visit was ordered, the physician could order subsequent visits, revise the care plan, or discharge the patient from the program. The CP PMO reviewed all the CP’s documentation and provided feedback for quality improvement.

### Referring Physicians

An online, 10-item survey was created and distributed to physicians who referred at least one patient to the CP program. The aim of the survey was to evaluate the physicians’ impressions of the program, communication with CPs, and overall satisfaction with the program.

### Data Analysis

We included for analysis patients enrolled from March 1–September 30, 2016. As part of data abstraction, the CP PMO eviewed patient health records to determine the primary medical reason for referral. Patients were grouped into one of three categories: high users needing medical management; high risk for readmission; and post-discharge follow-up. The study team exported all patient visits to the ED, all hospitalizations, and all primary clinic uses because primary care charges could result from an ED visit or hospitalization rather than from only a visit to the primary care physician’s office. The PMO evaluated all visits and clinic uses to determine whether they were related to the referring reason six months before enrollment and six months after enrollment. Although all patients were categorized with a single referring reason, most patients had comorbidities noted by the referring physician and were often referred with more than one reason. All visits and clinic uses were included for analysis if they were related to a referring reason. The Mayo Clinic Institutional Review Board approved this study.

### Objectives

The purpose of this study was to describe and evaluate the creation of a rural CP program and determine the change in healthcare utilization resulting from community paramedic in-home visits. The primary end-point was to analyze whether change in utilization type (ED, primary care, and hospitalizations) occurred by implementing CP visits. Further, we analyzed the number of CP visits required to create such reduction in utilization types.

## RESULTS

During the seven-month study period, 42 unique patients were enrolled in the program: 32 were classified as high users with medical management, six as high risk for readmission, and four as post-discharge follow-up.

### High Users Needing Medical Management

The median age of the 32 high users was 76 years; 22 (68.8%) were women. The total number of in-home CP visits for the six months after each patient’s enrollment was 412 (range, 1–47 per patient). Primary referral reasons are shown in Table 1. Primary care physicians referred seven patients (21.9%), emergency physicians referred 15 (46.9%), and hospitalists referred 10 (31.2%) as part of discharge from an admission.

Individual patient use of health services decreased from the six months before enrollment to the six months after enrollment (Table 2). The total number of visits and clinic uses decreased in the six months after enrollment (Table 3).

In the six months before enrollment, 10 patients required 911 services a total of 16 times. During the six months following enrollment, 10 patients had a total of 14 requests for 911 services. The payer mix for these 32 patients was 94% (30/32) government insurance (Medicare or Medicaid)/and 6% (2/32) private insurance.

### High Risk for Readmission and Post-discharge Follow-up

Six patients were categorized in the high-risk readmission group, but one patient was enrolled twice during the study period. Results of 72-hour and 30-day readmissions are shown in Table 4. The study team used the same outcome measures for the four patients enrolled for post-discharge follow-up. All patients were referred by a hospitalist before discharge or at discharge from a hospitalization.

### Referring Physicians

The survey for referring physicians garnered a response rate of 86% (18/21) (Table 5).

## DISCUSSION

Healthcare organizations, nationally, are looking for safe, high-quality mechanisms to get the right patient to the right place of care. In some cases patients can be managed in their homes with the right support and resources in place to avoid costly and potentially risky hospital admission. In the era of payment reform and many systems moving to an accountable care organization model, healthcare organizations look to mitigate readmission penalties and develop programs to manage patients remotely in their homes when possible.

Approximately 80% of older adults have at least one chronic disease, and over two-thirds of all healthcare costs are attributed to treating those diseases.^8^ The ability of these patients to self-manage varies. Patients who have difficulty with self-management may benefit from a CP program that is integrated with the patient’s primary care provider. A CP program can supplement clinic visits with physician-ordered vital sign monitoring, point-of-care testing, medication reconciliation, assistance in diet planning, and other areas of wellness.

The national, acute care 30-day readmission rate for Medicare beneficiaries is nearly 20%. Readmission rates greater than the national average put healthcare systems at risk for financial penalties from the Centers for Medicare & Medicaid Services.^9^ To decrease readmission rates, healthcare systems are developing methods to identify patients who have the greatest readmission risk. Organizations are turning to CP programs to help reduce that risk. A readmission can occur for various reasons, including adverse drug reaction, incorrect use of prescription medication, increased risk of fall, exacerbation of the primary cause for hospitalization, and poor wound care after a surgical procedure. Regardless of the reason, an integrated CP program can address these issues and more specific issues as identified and ordered by the physician.

While there was a reduction in primary care visits (n = 221), ED visits (n = 15) and hospitalizations (n = 9), there were a total 412 CP visits conducted to achieve these results. There was an increase in utilization when considering the addition of the CP visits. However, the cost of the CP visit compared to other visit types (primary care, ED, hospital) must be considered. In the case of primary care visit reduction, it is likely that these visits were merely replaced by the CP visit, which may have a cost benefit, especially to the patient, when considering patient travel and time away from work. Careful measurement of the cost of developing and deploying a CP program vs the savings from patient and payor expenses will be important for future research and a cost-benefit analysis.

Overuse of the ED can stress healthcare resources by increasing ED wait times, delaying ambulance response times, and diverting ambulances because of hospital crowding. Frequent patient use of the ED has been a long-standing issue,^10^–^15^ and patients who overuse the ED may also overuse other medical services, such as primary and inpatient care.^16^ Patients enrolled in the CP program for assistance in medical management and previous overuse of healthcare resources realized a decrease in primary care use, ED visits, and hospitalizations of 53.3%, 59.3%, and 60.0%, respectively. The decrease in use implies smaller charges to the patient and, given the primary payer sources of this population, a reduction in unreimbursed expenses to the health system.

This analysis was observational; however, several areas of future quality improvement were identified. Referring documentation lacked clearly defined patient care objectives, making it difficult to establish patient care and outcome goals to successfully discharge patients from the program. Future work will include implementing a care-planning process where the CP will create and document goals and objectives in conjunction with the patient and the primary care physician to create a plan for successful discharge from the program in the fewest visits necessary.

## LIMITATIONS

It was not possible to identify whether or when enrolled patients pursued medical care outside the health system. Accordingly, such use would not be represented in these findings. Further, healthcare providers were aware of this study, inherently introducing selection bias. While efforts were made to apply risk-assessment tools consistently, we could not control for a potential selection bias within the cohort. While we did observe decreases in clinic and hospital resource utilization, not all were statistically significant. It is important to continue to evaluate CP programs and publish results of large and diverse sample sizes. It is also necessary to account for the cost of start-up and maintenance of a CP program in comparison to the cost avoidance from ED and hospital utilization reduction. In this experience, 412 CP visits were conducted. Future programs will benefit from measuring and improving upon efficiencies where possible to provide the greatest impact with the fewest encounters. While decrease in utilization is described here, it must be acknowledged that CP visits themselves are a form of healthcare utilization and the number of visits conducted for this small sample size was extensive. The authors report no conflicts of interest. The authors alone are responsible for the content and writing of the paper.

## CONCLUSION

The implementation of a CP program targeting high users of healthcare resources resulted in a decrease in healthcare use in the hospital, ED, and primary care settings. This program may also reduce readmission rates for high-risk patients discharged from the hospital. Referring physicians generally agreed that the program benefited their patients.

## Figures and Tables

**Table 1:** Primary referral reason for patients categorized as high users of medical resources.

Primary referral reason	Patients, No. (%)
Falls	11 (34)
Chronic pain	6 (19)
Hypertension	4 (12)
Diabetes mellitus	3 (9)
Respiratory condition	3 (9)
Mental health	2 (6)
Multiple comorbidities	2 (6)
Congestive heart failure	1 (3)

**Table 2:** Individual patient use of health services before and after enrollment.

Health service	6-month period, number of patients		Difference^a^	
	
	Before enrollment	After enrollment	Patients, No.	Decrease, %
Primary care	30	14	−16 (p=.006)	53.3
Emergency department	27	11	−16 (p=.007)	59.3
Hospitalization	10	4	−6 (p=0.13)	60.0

a Difference = After enrollment - Before enrollment

Statistical Test: McNemar’s test of paired proportions was used to determine if there was a difference in the proportion of health services before and after enrollment. A continuity correction was applied to approximate the Chi-Square distribution.

**Table 3:** Aggregate use of health services before and after enrollment by the patient population (n=32).

Health service	6-month period, number of events		Difference^a^	
	
	Before enrollment	After enrollment	Events, No.	Change, %
Primary care	547	326	−221 (p<.001)	−40.4
Emergency department	60	45	−15 (p=.17)	−25.0
Hospitalization	16	7	−9 (p=.095)	−56.2
Community paramedic visits	0	412	NA	NA
Total healthcare contacts	623	790	+167	+26.8%

a Difference = After enrollment - Before enrollment

Statistical Test: For each health service, a z-test of proportions was used to test whether the number of tests before and after enrollment were the same. The z-test statistic was computed by comparing the proportion of tests for a given service that occurred after enrollment, and comparing to 0.5.

**Table 4:** Emergency department (ED) visit and readmission rates within 72 hours and 30 days after hospital discharge.

Patient category	≤72 Hours		≤30 Days	
	
	ED visit	Readmission	ED visit	Readmission
High-risk discharge (n=7), No. (%)	0 (0)	0 (0)	1 (14.3)	1 (14.3)
Postdischarge follow-up (n=4), No. (%)	0 (0)	0 (0)	0 (0)	1 (25.0)

**Table 5:** Physician survey results.

Survey item	Agree	Disagree	Undecided
I am comfortable with the community paramedic referral process	16	1	1
Patients I refer benefit from the community paramedic visit(s)	16	0	2
My expectations of the community paramedic visit(s) are met	17	0	1
Following a community paramedic visit, I see improvements in the patients’ health/wellness	14	0	4
Patients are satisfied with the care delivered by the community paramedic	18	0	0
I am satisfied with the ability to communicate with the community paramedic about care plans	17	0	1
The community paramedic is responsive to changes in the plan of care	14	0	4
The community paramedic provides quality care to the patients I refer	17	0	1
I would recommend this process to other clinicians	18	0	0
The community paramedic program should be expanded in my region	14	0	4
